# Extracellular Vesicles in Bladder Cancer: Biomarkers and Beyond

**DOI:** 10.3390/ijms19092822

**Published:** 2018-09-18

**Authors:** Yu-Ru Liu, Carlos J. Ortiz-Bonilla, Yi-Fen Lee

**Affiliations:** 1 Department of Urology, University of Rochester Medical Center, Rochester, NY 14642, USA; yu-ru_liu@urmc.rochester.edu (Y.-R.L.); Carlos_ortizbonilla@urmc.rochester.edu (C.J.O.-B.); 2 Department of Pathology and Lab Medicine, University of Rochester Medical Center, Rochester, NY 14642, USA

**Keywords:** extracellular vesicle, exosome, bladder cancer, biomarkers

## Abstract

Tumor-derived extracellular vesicles (TEVs) are membrane-bound, nanosized vesicles released by cancer cells and taken up by cells in the tumor microenvironment to modulate the molecular makeup and behavior of recipient cells. In this report, we summarize the pivotal roles of TEVs involved in bladder cancer (BC) development, progression and treatment resistance through transferring their bioactive cargos, including proteins and nucleic acids. We also report on the molecular profiling of TEV cargos derived from urine and blood of BC patients as non-invasive disease biomarkers. The current hurdles in EV research and plausible solutions are discussed.

## 1. Introduction 

**Figure 1:**
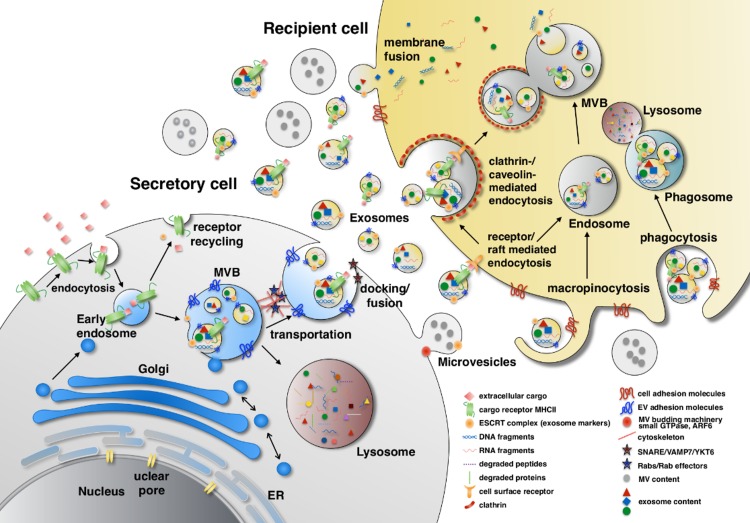
Extracellular vesicles (EV) biogenesis. The EV contents come from three sources: extracellular, intracellular and plasma membrane. Extracellular and plasma membrane molecules enter the early endosome through endocytosis either selectively by cargo receptor (ubiquitinated MHC-II) recognition or non-selectively. In the late endosome, the endosomal sorting complexes required for transport, ESCRT and their associated proteins such as TSG101, Alix, α-arrestin1 and CHMP4 mediate membrane inward invagination and form exosomes within multi-vesicular body (MVB). During the vesicle forming process, certain cytosolic components such as DNA, RNA and proteins are included in the exosome. MVBs can turn into lysosomes and degrade their contents or dock and fuse with the plasma membrane to release their contents to the extracellular space. The transportation and docking of MVBs is mediated by cytoskeleton remodeling which is regulated by Rab GTPase proteins (e.g., Rab27α, Rab27β and Rab7) and their effectors (e.g., SYTL4 and SLAC2B), whereas the fusion of MVBs with the plasma membrane is mediated by SNARE, VAMP7 and YKT6. In contrast, microvesicles are formed by outward budding of the plasma membrane which involves actin-myosin machinery, small GTPase and ARF6. The content sorting in microvesicles also involves TSG101. EV uptake is initiated by adhesion of EVs to the surface adhesion molecules on recipient cells, such as integrins, ICAM-1/LFA-1, CD11a, CD49d, CD44, CD169, heparin sulfate proteoglycans and by CD9, CD81 on EVs. EVs are then internalized through fusion, phagocytosis, macropinocytosis and endocytosis. ESCRT: Endosomal sorting complexes required for transport; TSG101: Tumor susceptibility gene 101; Alix: ALG-2-interacting protein X; CHMP4: Chromatin-modifying protein/charged multivesicular body protein; SYTL4: Synaptotagmin like 4; SLAC2B: Slp homolog lacking C2 domain B; SNARE: SNAP receptor; VAMP7: Vesicle associated membrane protein 7; YKT6: v-SNARE homolog (*S. cerevisiae*); ARF6: ADP-ribosylation factor 6; ICAM1: Intercellulare adhesion molecule 1; LFA1: Lymphocyte function-associated antigen 1.

In the past decade, a heterogeneous population of nanograde membrane particles in biological fluids, termed extracellular vesicles (EVs), gained newfound meaning in cancer therapy and diagnosis. EVs is a broad term which generally indicates the heterogeneous vesicles released from cells. In fact, most cells, if not all, shed vesicles constantly. Diverse names have been used to refer to various sorts of EVs, including ectosome, microparticle, exosome and microvesicle. Among them, the biogenesis, specific markers and functions of exosomes and microvesicles have been studied relatively thoroughly. As summarized in Figure 1, the release as well as uptake of EVs occurs simultaneously between cells. Exosomes are 50–100 nm in diameter and their biogenesis starts with the inward budding of a late endosomal membrane which forms a multi-vesicular body (MVB) containing a number of intraluminal vesicles (ILVs) [1]. In contrast, microvesicles (100–1000 nm in diameter) are larger than exosomes and formed by outward budding of the cell membrane. Both exosomes and microvesicles act as “intercellular postal service” [2] since they encapsulate a wide variety of bioactive molecules, including proteins, lipids and nucleic acids (DNA, micro-RNA, mRNA and other noncoding RNA species), and they transport this cargo to recipient cells locally or at a distance, consequently altering their behavior. The uptake of EVs by recipient cells is mediated through fusion, phagocytosis, macropinocytosis and receptor raft-mediated endocytosis. However, the mechanisms by which EV cargo is selected are not yet known. 

EVs gained biologists’ interest following the groundbreaking finding in 1996 that exosomes transfer Major Histocompatibility Complex (MHC) class II molecules from B cells to T cells, thus mediating activation of the adaptive immune response [3]. Later studies reported on the identification of various functional miRNAs encapsulated in EVs of immune cells. In view of the extensive regulatory capacity of miRNA, Valadi and colleagues in 2007 discovered for the first time that EVs have been exploited by cells as a tool to exchange genetic information [4]. This finding reveals a novel mechanism of gene-based communication between cells via EV cargo transfer. The pivotal roles of EVs are found not only in mediating the immune system but also in regulating various physiological and pathological cellular functions. The urinary bladder is susceptible to diverse EV-containing biological fluids, such as blood, lymphatic fluid and urine, reason why there has been an increased interest in EV roles in bladder cancer (BC) and study of their potential clinical applications. In this review article, we will focus on recent research on EVs derived from BC (BCEVs) and their roles in tumorigenesis and disease progression, as well as emerging applications in therapeutics and diagnostics.

## 2. Oncogenic Properties of BCEVs

Cancer cells are known to secrete more EVs than normal cells. The blood plasma of a cancer patient contains approximately 4000 trillion EVs, roughly twice the amount contained in a healthy individual [5]. Numerous studies have shown that EV-mediated cargo transfer to recipient cells affects many stages of cancer progression through communication between the cancer and the surrounding microenvironment, consequently promoting neoplastic transformation, BC proliferation, migration, invasion and angiogenesis. The EV cargo contents and their effects on cancer progression are summarized below.

### 2.1. BCEVs in Neoplastic Transformation 

The transformation of healthy cells into malignant cancer cells involves several pathologic processes and many studies indicate that TEVs participate by transferring oncogenic cargo molecules to recipient cells [6]. A study by Urciuoli et al. [7] reported that treating NIH3T3 fibroblasts with osteosarcoma-derived EVs induced tumor-like phenotypes. Cells gained survival capacity by enhanced proliferation, migration, adhesion and 3D sphere formation and acquired the ability to grow in an anchorage dependent manner. Similar findings were reported in a study by Panagopoulos et al. [8], where they showed that EVs isolated from DU145 prostate cancer cells induced the malignant transformation of non-malignant prostate epithelial cells, possibly via up-regulation of pro-survival protein STAT3 [9,10]. Together, these results demonstrate that TEVs promote malignant transformation. 

In the BC field, TEV’s role in tumorigenesis is less clear. Goulet et al. recently reported that BCEVs can promote “transformation” of healthy fibroblasts into cancer-associated fibroblasts (CAFs) [11]. They isolated EVs from RT4, T24 and SW1710 BC cells and used them to treat healthy fibroblasts isolated from human bladder biopsies. As a result, recipient fibroblasts gained CAF phenotypes with increased proliferation and migration capacity as well as elevated expression of CAF markers—smooth muscle actin (SMA), fibroblast activation protein (FAP) and Galectin. Interestingly, our unpublished data (12,24,60) reveal that chronically exposing non-malignant immortalized urothelial cells to BCEVs leads to malignant transformation in vitro and in vivo. This might be due to the selection of cells with resistance to a BCEV-induced cellular stress response [12].

### 2.2. BCEVs Promote Cancer Cell Progression by Mediating Communication between Tumor Cells

#### 2.2.1. Proliferation

The proliferation of tumor cells is an indispensable process for cancer progression, mostly relying on tumor-derived soluble growth factors. TEVs have been shown to promote cancer cell proliferation in leukemia, gastric cancer, glioblastoma, melanoma and prostate cancer, among others [13]. In BC, treating human 5637 and T24 BC cells with BCEVs was shown to stimulate their proliferation, possibly through activation of protein kinase B (Akt) and extracellular signal–regulated kinase (ERK) pathways [14]. Recent research delineating BC proliferation under hypoxia conditions found pivotal roles for BCEVs in transferring long non-coding RNA-urothelial cancer-associated 1 (lncRNA-UCA1) [15]. In this study, Xue et al. demonstrated that BCEVs derived from hypoxic 5637 cells contain high levels of lncRNA-UCA1 which stimulated proliferation, mobility and invasion in human UMUC2 BC recipient cells. In a xenograft model, lncRNA-UCA1-containing EVs facilitated bladder tumor growth and metastasis to the lymph nodes. Knockdown of lncRNA-UCA1 in hypoxic BCEVs increased the expression of E-cadherin while reducing vimentin and MMP9 expression, thereby triggering epithelial-mesenchymal transition (EMT) in the recipient BC cells.

#### 2.2.2. Migration and Invasion

The essential step of tumor progression to metastasis is gaining the ability to migrate and invade. Our previous study showed that EVs derived from high grade TCC-SUP BC cells as well as urinary EVs from patients with muscle invasive bladder cancer (MIBC) facilitated migration and invasion in low grade 5637 BC cells. Two TCC-SUP EV-enriched proteins, EGF-like repeats and discoidin I-like domain-3 (EDIL-3) [16] and periostin [17], were identified. They can activate the ERK1/2 MAP kinase signal pathway in recipient low grade BC cells, thereby promoting migration and invasion and knocking down EDIL-3 and periostin by shRNA disrupted this action. Similar results were reported by other group [18], which showed that EVs derived from T24 and UMUC3 BC cells enhanced urothelial cell migration and invasion. Also, blocking the EV uptake of recipient cells by heparin remarkably reduced BCEV’s impact.

In addition to carrying and transferring oncogenic cargos, BCEVs have been found to serve as an apparatus to dispose tumor-suppressor miRNAs (miR23b, miR224 and miR921) [19]. In this study, miRNAs previously identified to possess tumor-suppressor functions, such as miR23b, miR224 and miR921, were identified in BCEVs, implying a cancer character-sustaining mechanism. Silencing of Rab27α and Rab27β, two major EV secretion regulators, indeed halted the tumor-suppressing miRNA secretion. However, the miRNA retained in the cell might be inactivated by sequestration in the MVBs. Suppression of EV release resulted in reduced cellular invasion, which provides a possible explanation for the poor prognosis in BC patients with high expression of RAB27β. The levels of highly exocytosed tumor-suppressor miRNAs were found to be reduced in metastatic lymph nodes relative to primary tumors.

### 2.3. BCEVs Promote Cancer Cell Progression by Mediating Tumor-Stroma Communication 

The tumor microenvironment is composed of a complex and heterogeneous network of different cell types and the extracellular matrix (ECM). Tumor-associated stromal cells arise from various cellular origins: fibroblasts, pericytes, bone marrow mesenchymal stem cells, adipocytes and endothelial cells [20]. The communication between tumor cells and the tumor microenvironment is pivotal to both primary tumor growth and metastatic evolution and this is mediated through direct cell-cell contact as well as via tumor-secreted factors including EVs. One of the most characterized pro-cancer properties of TEVs is their ability to facilitate new growth in vascular networks within tumor microenvironments to sustain the rapidly growing tumor mass during metastasis. TEVs have long been known to be exploited to induce angiogenesis; however, the underlying mechanism was only revealed very recently in a breast cancer study [21]. TEVs derived from breast cancer MDAMB231 cells were reported to contain a unique vascular endothelial growth factor isoform, VEGF_90K_, that was crosslinked with Hsp90 and catalyzed by acyl transferase tissue transglutaminase (tTG). This EV-borne VEGF_90K_-Hsp90 complex stimulates tubulogenesis in HUVEC endothelial cells and this effect was diminished by the use of the HSP90 inhibitor 17AAG to force the release of VEGF_90K_ from the complex. Our group found that EVs from high grade BC cells contain EDIL-3 [16], which is known to promote tumor vascularization through an Arg-Gly-Asp (RGD) motif that interacts with integrin αvβ3 [22]. We demonstrated that the pro-angiogenic property of these BCEVs was abolished when EDIL-3 was suppressed by shRNA, confirming that EV-borne EDIL-3 mediates recipient endothelial angiogenesis.

Another key event mediated by TEVs during cancer progression is the establishment of a pre-metastatic niche (PMN) in favor of future circulating tumor cell (CTC) adhesion and colonization, which eventually leads to metastatic outgrowth. Growing evidence indicates that TEVs play central roles in PMN establishment and maintenance processes such as vascular remodeling, immune modulation, metabolic environment modification, fibroblast differentiation into CAF, ECM re-organization and organotropic homing [23]. However, the difficulty of obtaining pre-metastatic tissues from cancer patients and the lack of metastatic BC animal models have limited clinical investigation into the significance of this phenomenon. Our laboratory has succeeded in isolating metastasis-prone MB49 sub-lines and we have found that pre-conditioning mice with sub-line EVs promotes lung metastases (manuscript in preparation). A broad panel of ECM components is enriched in MB49 sub-line EVs, suggesting that they may participate in PMN formation principally through ECM re-organization [24]. 

## 3. Regulation of Immune Responses by BCEVs 

Recent global profiling of the genetic and epigenetic landscape of BC has revealed it to be one of the most mutated cancers after lung cancer and melanoma [25,26]. Many new mutations have been identified; interestingly, many of them coincide with mutations that have been discovered previously in BC. This demonstrates that progressive tumors are heterogeneous, making it difficult to predict their outcome and the signatures of some of these molecular alteration patterns seem to have a prognostic impact [27]. With such a high mutation rate, BC can produce many tumor-associated antigens (TAAs) that are either mutated cellular proteins or molecules with different post-translational modifications [28]. The formation of TAAs leads to the generation of TAA-derived peptides, which are then presented through MHC on the surface of cancer cells to activate immunological surveillance. Since EVs have been known to modulate immune responses by directly or indirectly presenting MHC-antigen peptide complex on their surface, it is likely that these TAA-derived peptides can also be loaded into BCEVs to mediate immune response. In this section, we will discuss BCEVs functional roles in regulating the immune system. 

### 3.1. Immune System Activation by BCEVs 

While the activation of the immune system by cancer cell-derived EVs is not a well-studied phenomenon, there are a few reports that support this claim. For example, Rao et al. reported that TEVs elicited an antitumor immune response in a murine hepatocellular carcinoma (HCC) model in vivo [29]. They isolated TEVs from the murine HCC cell line hepa1-6 and used them to activate DC2.4, a murine dendritic cell (DC) line. These TEV-pulsed DCs were orthotopically injected into HCC tumor-bearing C57BL/6 mice, which resulted in increasing infiltration of T lymphocytes and elevated levels of interferon-γ (IFN-γ), consequently suppressing tumor growth. A similar finding was reported by Bu et al., who found that TEV-pulsed DCs elicited a tumor-specific CD8^+^ cytotoxic T cell response in glioma patients [30]. In this study, they applied patient-derived T cells and CD14^+^ DC precursor cells and found that EVs from the tumors of the same patients can activate T cell-mediated cytotoxicity. In the context of BC, Zhang et al. found that BCEV-educated DCs elicit T cell cytotoxic activity in vitro [31]. This evidence supports the possibility that BCEVs can promote immune system activation to facilitate the anti-tumor immune response. 

### 3.2. Immune System Suppression by BCEVs

TEVs are known to be able to suppress the immune surveillance system, allowing tumor cells to escape the immune barriers and grow. This role of TEVs has been extensively studied using various cell types involved in the immune surveillance of tumors. In one immune escape strategy, cancer cells downregulate their MHC class I surface expression. However, natural killer (NK) cells are known to recognize and eliminate those non- or low-expressing MHC class I cells [32], so as a defense mechanism cancer cells can secrete EVs bearing transforming growth factor β1 (TGFβ1) to deactivate NK cells and decrease their cytotoxic activity, resulting in the suppression of the anti-tumor immune response [33]. 

Shinohara et al. reported that the presence of miR145 in colorectal cancer TEVs can polarize classic (M1) type macrophages into M2 type macrophages, thereby supporting cancer cell growth in vitro and in vivo [34]. Further mechanistic dissection revealed that miR145 directly binds to the 3’untrasnlated region (UTR) of *HDAC*II, a histone deacetylase, silencing its expression and promoting interleukin 10 (IL-10) production. 

TEV suppression of DC function was demonstrated by Salimu et al. [35]. They treated DC cells with TEVs isolated from DU145 prostate cancer cells and co-cultured them with CD8^+^ T cells. TEV-educated DCs triggered significantly stronger tumor-antigen-specific T cell responses as determined by IL-2 and IFN-γ production. 

TEVs also allow immune escape by inactivating T lymphocytes directly. Rong et al. discovered that breast cancer cells secrete TEVs capable of suppressing T lymphocytes [36]. A similar phenomenon was found in head and neck cancer patients, where TEVs suppressed T lymphocytes, allowing tumor progression [37].

In BC, an important question that remains unanswered is whether EVs have an immunosuppressive character as seen in other cancer types. Last year, Lee et al. found that EVs derived from BC patient urine present an altered protein composition [38]. They found significant upregulation of mucin-1 (MUC1), carcinoembryonic antigen (CEA) and moesin. MUC1 has been reported to contribute to NK cell evasion by cancer cells [39] and its expression level has been associated with BC prognosis [40]. CEA has been correlated with tumor angiogenesis [41] and can inhibit NK cell targeting of cancer cells [42]. Moesin has been associated with metastasis and poor prognosis in a number of different cancers, including pancreatic, colon and laryngeal carcinomas [43,44,45,46]. These findings suggest that BCEVs might have immunosuppressive roles and open a new avenue for future research.

### 3.3. BCEVs in Promoting Inflammation

BCEVs may also have a role in controlling inflammation. We reported that MIBC patient urinary EVs are enriched in transaldolase (TALDO1) [47], an enzyme linked to oxidative stress, inflammation and carcinogenesis [46]. ApoB is another BCEV protein with a functional link to the inflammation process [48]. ApoB is another BCEV protein with a functional link to the inflammation process [49]. Andreu et al. compared the urinary EV protein profiles of BC patients versus healthy non-smokers and found that ApoB expression was significantly increased in BC patient-derived EVs. ApoB is involved in a wide range of biological processes including secretion associated with exosomes [50] and EVs [51]. ApoB has also been reported to play important roles in angiogenesis [52] and inflammation [53]. 

In summary, our understanding of BCEVs’ functional roles in regulation of immune response is still in its initial stage. With recent progress made in cancer immunotherapy and the emerging evidence of BCEVs mediating communication between tumor and immune cells, we anticipate that further research will reveal pathological roles of BCEVs and their cargos in the regulation of immune responses, especially in response to checkpoint inhibitors. 

## 4. Therapeutic Application of BCEVs

### 4.1. EV-Mediated Delivery of Therapeutic Agents in BC

Nanomedicine was introduced in cancer therapy during the 1990s [54]. With the benefit of small size (usually less than 200 nm), nanoparticles are able to escape from being engulfed by macrophages and neutrophils (which eliminate particles about 250–1000 nm) and then diffuse into the blood circulation and be transported to their target sites. With EVs’ small size, various cell origins and low cytotoxicity, EVs have become an ideal nanoparticle drug carrier [55]. 

EVs were first used as a drug delivery vehicle to transport curcumin, an anti-inflammatory drug, to treat brain inflammatory disease [56]. Administration of exosomes encapsulating curcumin resulted in 5–10 fold higher plasma concentrations than curcumin alone and more effective inhibition of LPS-induced brain inflammation. BC cells are known to take-up EVs in a dose-dependent manner [57]. A recent study also found robust EV internalization in BC cells [58] where human BC cell lines (SW780 and UMUC3) showed 20–50 fold higher EV internalization rates than normal urothelial cells. Such high uptake rates make EV-nanoparticles an attractive method of drug delivery to BC cells. Moreover, the membrane structure of EVs encapsulates and protects vulnerable molecular contents, in particular various RNA species, such as siRNA, miRNA and lncRNA. In a recent study, EVs were exploited as a vector to deliver the designed siRNA to BC cells [58]. EVs were loaded with artificially synthesized siRNAs targeting polo-like kinase-1 (PLK1) by electroporation and then used to treat UMUC3 cells. As a result, the UMUC3 expression of PLK1 was significantly decreased, consequently inducing apoptosis and necrosis. 

Chemotherapy following removal of the primary tumor is the standard treatment in many cancers. While chemotherapy is often capable of inducing cell death in tumors, many patients develop more advanced tumor growth due to the appearance of chemo-resistance, which remains one of most challenging problems in cancer research today. A recent study reported an innovative approach of using TEVs to sensitize BC cells to chemotherapeutic agents [59]. In a mouse model, intravesical instillation of TEVs prior to instillation of drugs including doxorubicin, mitomycin C, hydroxycamptothecin and gemcitabine, significantly reduced hematuria and tumor incidence. These TEVs were initially collected from UV-treated tumor cells and ranged in size from 100–1000 nm (microparticles). The recipient BC cells internalized the EVs into lysosomes, increasing lysosomal pH from 4.6 to 5.6, thereby promoting transportation of the lysosome to the nucleus over exocytosis and subsequently retaining drug bioactivity in the BC cells. 

In the context of immunotherapy for BC, our group found that Bacillus Calmette–Guérin (BCG) infection stimulated BC cells to release EVs that could activate T lymphocytes, bone marrow-derived DCs and macrophages in vitro. This unpublished data suggests that TEVs are capable of mediating the anti-tumor immune response, possibly from transferring immune-active cargos [60].

### 4.2. Prognosis and Diagnosis of BC Using EVs 

There is a growing trend towards exploring the use of minimally invasive liquid biopsy for early cancer detection and TEVs are attractive sources of cancer diagnostic and prognostic biomarkers for the following reasons: (1) EVs contain a specific cargo of proteins and RNAs that might reflect the status of the originating cells, (2) EVs are membranous structures that can protect the cargo contents from degradation, [61] EVs are relatively accessible as they are found in clinical specimens that can be obtained through non-invasive methods. Apart from plasma/serum, urine is considered the most relevant body fluid in terms of its physical contact with bladder tumor mass. Although EVs compose only 3% of excreted urinary protein [62], with proper isolation methodology and proteomic analysis, many urinary exosomal proteins have been identified to have pathophysiologic significance [61,63,64,65,66,67,68,69]. Nawaz et al. in 2014 published a comprehensive review of EVs as biomarkers for urogenital cancers which addressed the great potential of utilizing EVs in prognosis and diagnosis [70].

To define appropriate baselines, proteomic investigation of EVs derived from healthy donors is needed. The first comprehensive study of urinary EV protein contents was performed by Pisitkun et al. in 2004 using liquid chromatography-tandem MS (LC-MS/MS) [71]. Soon after, more detailed proteomic analyses were reported which determined protein profiles for urinary EVs of bladder and prostate gland origin [68,72,73,74,75,76].

Cell-free urine has been used to predict treatment response, recurrence, prognosis and diagnosis by detecting DNA level, methylation, mutation and integrity [77,78]. In BC, DNA level and integrity in cell-free urine were found to be significantly elevated relative to controls [79,80,81]. Urinary EV profiling of quantity as well as miRNA and protein content has been reported to serve as a prognostic and diagnostic biomarker. Recently, Liang et al. developed an integrated double-filtration microfluidic device to measure EV concentration at the point-of-care. They found higher amounts of EVs in the urine of BC patients compared to healthy controls and this result further suggests that urinary EVs have great potential to be used as a disease biomarker for BC [82]. Profiling miRNAs in cell-free urine was demonstrated to have >80% sensitivity and specificity in detecting different stages of BC [83]. Proteomic analysis of urinary EV cargo provides another prospect for disease prediction. Lin et al. collected urine EVs and analyzed the proteomic data from 129 BC patients versus 62 healthy participants and found SERPINA1 and H2B1K as promising BC biomarkers for prognosis Proteomic analysis of urinary EV cargo provides another prospect for disease prediction. Lin et al. collected urine EVs and analyzed the proteomic data from 129 BC patients versus 62 healthy participants and found alpha-1 antitrypsin (SERPINA1) and Histone H2B type 1-K (H2B1K) as promising BC biomarkers for prognosis [84]. We have searched the cargo contents of EVs derived from BC cells and urine of BC patients from the past 10-year publication and summarized the list of miRNAs and proteins encapsulated in EV cargos in Table 1 and Table 2, respectively. The BC patient urinary EVs are a mixture of the whole body EVs and BCEVs, which reflects the clinical reality and relevance. Note that most of the reported cargo molecules are based on global screening that identified differentially displayed miRNAs and proteins between BC samples and controls but their functional roles in BC have not been verified. 

## 5. Current Challenges and Future Prospects

### 5.1. Current Challenges 

Researchers have used dozens of names for various secreted vesicles (including exosomes, microvesicles and EVs), which have been broadly used and are sometimes interchangeable. However, exosomes and microvesicles are functionally and structurally distinct; there are differences in charge, size and molecular composition [98]. Importantly, the size distributions of exosomes and microvesicles overlap significantly and the identity of EVs between 100–150 nm in diameter is ambiguous [12]. Therefore, size alone cannot always be used to distinguish these EV subpopulations from one another. While “extracellular vesicle” is a widely accepted generic term for all secreted vesicles, there is a need for consensus about how to apply the other terms appropriately to different EV subpopulations in terms of vesicle size. 

The conflicting names for different EV subpopulations are largely due to the different procedures used in individual laboratories to obtain and sort biological fluids to isolate EVs. Currently, with the rapid increase in the understanding of EV biology, including their function in numerous aspects of human disease and their potential significance in clinical applications, there is a growing demand for simple, efficient and reliable techniques to isolate EVs. Until now, the most standard EV isolation procedure combines filtration and ultracentrifugation, which purify particles based on their size and density [99]. To further purify exosomes from EVs, a common technique uses a continuous sucrose gradient during ultracentrifugation, which distributes particles according to density (exosomes float at densities ranging from 1.15–1.19 g/mL) [100]. In addition, microfluidic techniques combining immune-affinity, sieving and trapping have been applied to concentrate exosomes [101,102,103]. However, the unavoidable damage to the exosome structure and the low recovery narrows the application of this technique. Another common EV isolation method that has also been widely used for exosome purification is immune-affinity precipitation. This technique captures exosomes using antibodies against exosome surface markers. However, this method is limited by the exclusion of some EV subpopulations that do not carry the well-known markers. Therefore, the identification of general markers for EVs, such as lipid composition, pH value and electrical properties might be useful for capturing whole EV populations [104]. With the rapidly growth of the field, more and more isolation methods are proposed, the most updated EV isolation technic were comprehensively covered by recent reviews [1,12]. The recent launched EV-TRACK database encourages researchers to report their EV isolation details for developing a standardized protocol. (http://evtrack.org).

One of the hurdles to urinary EV isolation is the aggregation of highly abundant non-exosomal proteins, such as Tamm-Horsfall protein (THP), which tends to form fibrillary aggregates at low temperature. This aggregation during the EV isolation process was proposed to be reduced by a disulfide bond reducer, such as dithiothreitol (DTT), or a mild solubilizing detergent, such as CHAPS (3-[(3-cholamidopropyl) dimethylammonio]-1-propanesulfonic), which can separate THP from EVs during differential centrifugation [99,105,106,107]. However, DTT treatment can cause changes in the extracellular domains of EV proteins that would affect their stability and function. CHAPS treatment is better at preserving EV features but requires longer preparation [99]. 

The major challenge of EV-based biomarker discovery is the lack of a validated and standardized approach to normalize body-fluid concentrations among patients, especially in urine samples due to variation of water excretion in each individual. Urinary creatinine (UCr) excretion in the renal system is considered to be constant across and within individuals and is commonly used to normalize urinary biomarker concentrations against variations in urine flow rate in the evaluation of chronic kidney disease and prediction of acute kidney injury [108,109]. However, creatinine excretion rates vary widely among individuals with different age, sex, race, diet, physical activity, muscle mass, emotional stress and disease state [110,111], thus potentially masking the true value of EV proteins. Alternatively, specific exosome markers such as TSG101 and Alix can be used for normalization of urinary EV proteins [112]. More studies are needed to evaluate these normalization techniques and/or identify new ones. 

Urinary EVs originate from cells throughout the urinary system; therefore, it is important to distinguish BC-specific EVs from the heterogeneous population of urinary EVs shed from other sources such as kidney and prostate. A recent study was able to increase the purity of podocyte-derived exosome isolation using immune-absorption with antibodies against the podocyte-specific complement receptor type 1 (CR1). Proteomic analysis of the podocyte EVs identified 14 new podocyte EV-enriched proteins that can potentially be used as kidney-specific EV markers to distinguish them from the broader urinary EV population [113]. This finding encourages similar efforts to identify BC-specific EV markers that are greatly needed to improve the diagnostic utility of urinary EVs. 

### 5.2. Future Prospects 

With accumulating evidence of TEVs’ functional roles in cancer progression, depletion of the TEVs in circulation while retaining normal and healthy EVs becomes an ideal therapeutic approach. In 1989, Lentz conducted a primary experiment to remove low molecular weight (<120 kDa) proteins from cancer patients’ blood by ultrapheresis, which resulted in tumor size reduction in 6 out of 16 patients [114]. At that time, serum cytokine receptors were proposed to be the key factors in blocking the antineoplastic immune response. However, this therapeutic effect might be because the process also results in the elimination of EVs. Previously, plasmapheresis combined with an affinity matrix containing *Galanthus nivalis* agglutinin to capture hepatitis C viruses has been applied clinically [115]. A similar plasmapheresis system was adapted to capture TEVs using a specific antibody-conjugated cartridge [116]. Therefore, identifying TEV-specific surface markers is the crucial step to take this approach to the next stage. 

Another TEV targeting strategy is the inhibition of EV biogenesis and uptake. Amiloride, an endocytic vesicle recycling inhibitor, reduces the EV amount in the circulation and increases chemotherapy effects in mice [117]. Interference with the key proteins in EV biogenesis, such as Rab27β, also results in inhibition of EV release and reduction of tumor progression [118,119]. Theoretically, inhibiting EV uptake can be achieved by blocking surface phosphatidylserine. However, such inhibition can also affect microvesicle uptake by normal cells that might cause off-target side effects. Further dissection of EV machinery might lead to the identification of regulatory pathways in EV biogenesis or internalization that are specifically utilized by cancers. 

The mechanisms by which secreted EVs are targeted to recipient cells are not yet well understood. It has been suggested that various integrins expressed on the surface of EVs might determine that they will interact with specific recipients through ligand-receptor binding [56,120,121]. A study by Hoshino et al. found that EVs from a variety of cancer cell types were preferentially taken up by specific cells in various organs depending on their integrin expression [122] This finding raises the possibility of utilizing EVs as therapeutic vectors to deliver RNA, protein or drug cargos to specific targeted cells by genetically engineering the EV integrins [123]. As more understating of the physical and pathological role of EV, more applicable areas of BCEV will be proposed. 

## 6. Conclusions

**Figure 2:**
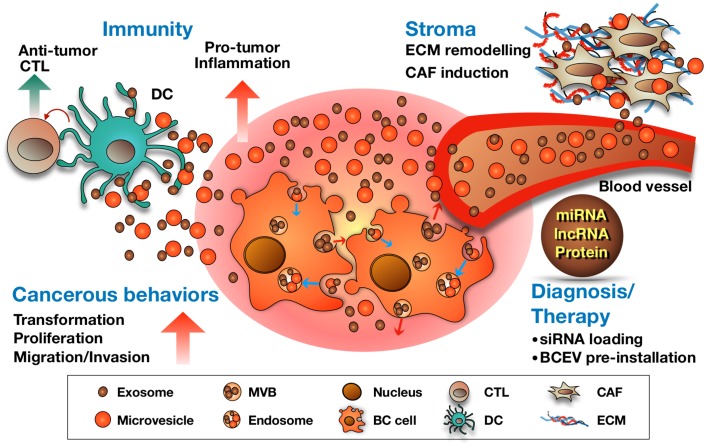
Summary of the roles of BCEVs in cancer, the tumor microenvironment and therapeutic applications. BCEVs are involved in many aspects of cancer development and progression. Like other cancer cells, BC cells release EVs into extracellular spaces and can be received by urothelial cells and immune cells, consequently modifying their behavior to support or suppress tumor growth (red and blue arrows indicate the migrating direction of intracellular vesicles). On the one hand, BCEVs can promote neighboring recipient cells’ cancerous behaviors, including malignant transformation, proliferation, migration and invasion, as well as modify the tumor microenvironment in favor of tumor outgrowth, including promoting inflammation, ECM remodeling and fibroblast differentiation to cancer-associated fibroblasts (CAF). In contrast, BCEVs also participate in the immune surveillance system by presenting tumor antigens to provoke dendritic and cytotoxic T cell anti-tumor immunity. With specific cargoes carried by BCEVs such as miRNA, lncRNA and proteins, their clinical application, particularly in disease biomarkers, has rapidly expanded. Moreover, researching the utilization of BCEVs as vesicles to deliver therapeutic materials is also underway.

In this review article, we have discussed various functional roles of BCEVs in mediating BC pathogenesis. As summarized in Figure 2, BCEVs can drive normal urothelial cell malignant transformation, promote BC progression via stimulation of proliferation, invasion and migration of recipient neighboring BC cells and modify the tumor stroma to support tumor growth. BCEVs have been further suggested to have roles in mediating cancer-related immunity, either by promoting inflammation favorable to tumors or by participating in the immune surveillance mechanism. Finally, potential clinical applications of BCEVs, mainly in diagnosis or prognosis or as drug-delivery vehicles, are discussed. However, the normal physiological functions of EVs should not be neglected, so that the off-target side effects of EV-based therapy can be reduced. As to EV-based liquid biopsy development, the identification of tissue/disease-specific EV markers is necessary to facilitate sorting of TEVs from the heterogeneous EV populations in patient specimens. Further investigation of EV biogenesis, content packing and uptake is also critical for future applications.

## Figures and Tables

**Table 1:** List of miRNAs identified in BC urinary EVs and/or BC cells EVs.

miRNA	Regulation	Sample Sources	Reference
miR-21	up	urine & BC cells lines	[85,86,87,88,89]
miR-200c	up	urine	[85,86,88]
miR-23b	up	urine	[19,90]
miR-513b-5p	up	urine	[90,91]
miR-183	up	urine	[88,92]
miR-205	up	urine from NMIBC patients	[86,88]
miR-16-1-3p, miR-28-5p, miR-92a-2-5p, miR-142-3p, miR-195-3p, miR-196b-5p, miR-299-3p, miR-492, miR-601, miR-619-5p, miR-3155a, miR-3162-5p, miR-3678-3p, miR-4283, miR-4295, miR-4311, miR-4531, miR-5096, miR-5187-5p	up	urine	[90]
miR-155-5p, miR-132-3p, miR-31-5p, miR-15a-5p	up	urine	[87]
miR-93, miR-940	up	urine	[85]
miR-16, miR-96	up	urine	[92]
miR-486-5p, miR-205-5p, let-7i-5p	up	urine from NMIBC/(G1 + G2)	[88]
miR-106b-3p, let-7c-5p, miR-486-5p, miR-151a-3p, miR-200c-3p, miR-183-5p, miR-185-5p, miR-224-5p	up	urine from NMIBC/G3	
miR-4454, miR-720/3007a, miR-29-3p	up	urine from NMIBC	[86]
miR-214	up	urine from NMIBC	[93]
miR-503-5p, miR-145-5p, miR-3158-3p, miR-30a-3p	up	urine from MIBC	[91]
miR-106b-3p, miR-486-5p, miR-205-5p, miR-451a, miR-25-3p, miR-7-1-5p, miR-146a-5p	up	urine from MIBC	[88]
miR-1, miR-99a, miR-125b, miR-133b, miR-143, miR-1207-5p	down	urine	[92]
let-7f-2-3p, miR-520c-3p, miR-4783-5p	down	urine	[90]
miR-30c-2-5p, miR-30a-5p	down	urine from NMIBC/(G1 + G2)	[88]
miR-30a-5p, miR-30c-2-5p, miR-10b-5p	down	urine from NMIBC/G3	
miR-30a-5p, let-7c-5p	down	urine from MIBC	
miR-27b-3p	down	BC cells	[91]
miR-let-7i-3p	down	BC cells	[89]
miR-29c-5p, miR-146b-5p, miR-200a-3p, miR-200b-3p, miR-141-3p	down	BC cells	[91]

**Table 2:** List of proteins identified in BC urinary EVs and/or BC cells EVs.

Protein ID	Sample Sources	Validated	Proteomic Detection
EHD4	urine and BC cells	[47]	[16,38,94]
HEXB	urine and BC cells		[16,38]
ANXA; SND1	urine and BC cells		[16,95]
S100A4	urine and BC cells		[16]
TALDO1	urine and BC cells		[16]
MUC1	urine and BC cells	[38,96]	[95]
EPS8	urine	[38]	[94]
CEAM5	urine		
CD44; BSG	BC cells	[96]	
ITGB1; ITGA6; CD36; CD73; CD10; CD147; 5T4	BC cells		
NRAS; MUC4	urine	[94]	
SERPINA1 H2B1K	urine	[84]	
TACSTD2	urine	[74]	
EDIL3	urine and BC cells	[16]	
POSTN	urine and BC cells	[17]	
CTNNB1; CDC42	urine and BC cells		[95,97]
14-3-3; ALIX; B2M; EGFR; EZR; FSCN1; LGALS; GST; MSN; PRDX1; PTGFRN; RDX; TAGLN2	BC cells		[95]
